# Production of polyhydroxybutyrate by coupled saccharification–fermentation of inulin

**DOI:** 10.1007/s00449-023-02953-7

**Published:** 2023-11-25

**Authors:** Fernando Guzmán-Lagunes, Lorena Martínez-dlCruz, Phavit Wongsirichot, James Winterburn, Carmina Montiel

**Affiliations:** 1 Food Sciences and Biotechnology Department, Faculty of Chemistry, Universidad Nacional Autónoma de México, Mexico City, Mexico; 2 Department of Physical Chemistry, Faculty of Chemistry, Universidad Nacional Autónoma de México, Mexico City, Mexico; 3 Department of Chemical Engineering, Faculty of Science and Engineering, The University of Manchester, Manchester, UK

**Keywords:** Enzymatic hydrolysis, Inulin, Polyhydroxybutyrate, *Cupriavidusnecator*, Bioplastics

## Abstract

Inulin is a fructose-based polysaccharide that can be found in several plant species, from grass and onions to chicory roots; thus, it has the potential to be an excellent renewable source of fructose for several industrial applications. Among them, inulin hydrolysis can be coupled to a fermentation operation to produce polyhydroxybutyrate (PHB) using *Cupriavidus* *necator* H16. This work reports the PHB production process involving chicory root inulin hydrolysis using inulinase Novozym 960 followed by a *C.* *necator* fermentation. It was found that the maximum saccharification (95% wt.) was reached at 269 U/g_inulin_ after 90 min. The hydrolysates obtained were then inoculated with *C.* *necator,* leading to a biomass concentration of 4 g/L with 30% (w/w) polymer accumulation. Although PHB production was low, during the first hours, the cell growth and polymer accumulation detected did not coincide with a fructose concentration decrease, suggesting a simultaneous saccharification and fermentation process, potentially alleviating the product inhibition inherent to the inulinase–fructose system. The characterization of the obtained PHB showed a polymer with more homogeneous values of *M*_w_, and better thermal stability than PHB produced using pure fructose as a fermentation substrate. The results obtained demonstrate a viable alternative carbon substrate for PHB production, opening the possibility for inulin-rich renewable feedstock valorization.

**Supplementary Information:**

The online version contains supplementary material available at 10.1007/s00449-023-02953-7.

## Introduction

Inulin is a fructan consisting of β-2,1-linked fructose residues joined to a chain-end glucose, it is synthesized by several plant species as carbon and energy reserves and is one of the most abundant polysaccharides on the planet [1]. Inulin molecules of up to 70 fructose units can be found in many plants, such as chicory and Jerusalem artichoke, which are currently used as feedstocks for inulin industrial production, although it can be found in several others, i.e., grass, onions, and agave. The industrial exploitation of some of these plants generates a surplus of inulin-rich wastes, available for use as feedstock in different regions of the world [2]. The growing interest in inulins and their derivatives obtained from hydrolysis has triggered the development of different valorization strategies and the necessary processes to allow for the holistic exploitation of such residues [1, 2].

Inulin hydrolysis leads to high-value products with a wide range of applications including high-fructose syrup and fructooligosaccharides (FOS). Compared to chemical treatments, the enzymatic hydrolysis of inulin offers the advantages of specificity, avoiding the formation of undesired by-products, and the reduction of the energy requirements of the process [3]. Exoinulinases degrade inulin from its non-reducing end, releasing fructose molecules, while endoinulinases hydrolyze the internal β-2,1-glycosidic linkages arbitrarily, producing FOS of various chain lengths [1, 4]. Furthermore, fructose produced by exoinulinases can be used in the food industry as fructose syrup or as a potential platform for biotechnology production of added-value chemicals through different fermentation processes [5, 6].

One potential strategy for the valorization of fructan-rich residues is their use as feedstock for polyhydroxyalkanoates (PHAs) production. PHAs are bio-based, biodegradable, and biocompatible polymers with similar mechanical and material properties to those of the commonly used plastics derived from petroleum [7]; thus, PHAs have been proposed as a sustainable solution to the ecological impact that plastic accumulation has on the environment. Nevertheless, despite the environmental advantages offered by their wider application, the high production cost of PHAs continues to hinder their industrial implementation [8]. The two main contributors to the overall PHA production costs are the utilization of purified carbon sources and the downstream purification process of the polymers from the biomass matrix, each representing around 40% of the total cost [9, 10]. Therefore, using cheaper raw materials has been proposed as a strategy to reduce PHA production costs. Depending on the regional produce and availability, many cheap raw materials have been studied as feedstock for PHA production, including food and dairy wastes, lignocellulosic biomass, and waste oil, amongst others [11–15]. Different microorganisms and microorganism-consortia have been studied as potential PHA producers, such as *Bacillus* *sp*, *Pseudomonas* *sp*, *Burkholderia* *sp,* *Loktanella* *sp*, *Azohydromonas* *lata,* *Halomonas* sp, *Haloferax* *mediterranei*, and *Cupriavidus* *necator* (previously known as, *Ralstonia* *eutropha*) [16–18].

*Cupriavidus* *necator* is the most studied bacterium for PHA production due to its capacity to accumulate up to 80% (w/w) of its dry matter in polyhydroxybutyrate (PHB), the most common PHA, using fructose as a carbon source [19]. Nevertheless, developing a production method to obtain a fructose-rich medium that this bacterium can utilize is key to establishing a sustainable industrial process [20].

This work aims to determine the proof of concept and technical feasibility of the use of chicory inulin, a commercially available fructose-rich material, as feedstock for PHA production, using an inulin enzymatic hydrolysis process to produce a fructose-rich medium that can subsequently be used as a substrate by *C.* *necator*. A coupled inulin saccharification and fermentation process was studied for the production of PHB, where fructose is freed from the inulin matrix into the reaction medium and assimilated by the microbial strain to produce PHB. Thus enabling a valorization strategy for inulin-rich substrates available worldwide towards the production of an alternative bio-based and biodegradable substitute to plastics.

## Materials and methods

### Enzymatic hydrolysis of inulin

Commercial Inulinase Novozym 960 (Novozymes, Denmark) was used to hydrolyze inulin. To explore the feasibility of producing PHA from enzymatic hydrolysates, chicory inulin (Sigma-Aldrich, USA) was selected as the substrate to work as a model for the hydrolysis reaction due to its linear structure and commercial availability. An initial inulin concentration of 5 g/L was set for the analysis during the first stage. Initial enzyme concentration was varied from 1 to 5% (v/v) to determine the best ratio enzyme substrate for the hydrolysis process.

The reactions were performed in a total volume of 500 μL acetate buffer, pH 5, 0.05 M, at 50 °C, the best conditions evaluated for this enzyme by our group using inulin from chicory as substrate [21]. Different volumes of Novozym 960 (44.88 U/mL enzyme), from 5 to 25 µL, were added to start the hydrolysis reaction. The enzyme activity was measured by calculating the inulin-to-fructose conversion. *U* is defined as the amount of enzyme required to produce μmol fructose equivalent per minute under standard assay conditions.

Reactions were run for 3, 9, 10, 20, 30, 40, 50, 60, and 90 min and stopped by heating the mixture at 90 °C for 5 min. Carbohydrate determination by the DNS method was conducted for the samples obtained. All experiments were performed in triplicate.

Subsequently, the effect of the substrate concentration on the enzyme activity was evaluated under the reaction conditions described above; initial inulin concentrations assayed were 5, 10, 15, 20, 25, and 30 g/L, maintaining the enzyme:substrate ratio at 269.28 U/g_inulin_. Reactions were stopped at 20, 30, and 40 min; carbohydrate determination was performed to each sample.

### Product inhibition assay

Initial fructose concentrations of 10, 15, 20, and 25 g/L were added to the hydrolysis medium to evaluate the effect of the reaction product on the enzymatic activity. Reactions were run for 40 min to maximize the fructose yield of the hydrolysis, and the initial inulin concentration was set to 10 g/L. Released sugars were measured to evaluate the saccharification yield of each system.

### Hydrolysates fermentation

*Cupriavidus* *necator* *H16* was purchased from the American Type Culture Collection, ATCC 17699. A glycerol 40% (v/v) stock culture was prepared using the rich media recommended by the supplier, containing beef extract 3 g/L and peptone 5 g/L (Bioxon, Mexico), with a new cryovial being used to begin each separate experiment. Adding agar, 15 g/L, to the rich medium preparation was necessary for short-term storage. Cells were activated using the rich medium described before. After 24 h of growth, cells were used to inoculate a seed culture using the medium described by Aramvash et al*.* (2015) containing KH_2_PO_4_ 1.75 g/L; MgSO_4_⋅7H_2_O 1.2 g/L; citric acid 1.7 g/L; NH_4_Cl 2 g/L (J. T. Baker, Mexico) with the addition of 10 g/L fructose as a carbon source [22]. 4 M solutions of NaOH and HCl were used to adjust the pH of the medium to 6.8. Mineral salts and fructose solutions were sterilized separately and mixed afterward, under aseptic conditions. Incubation conditions were set at 30 °C and 250 rpm.

When the seed culture reached 23 h, parallel inulin hydrolysis reactions of 50 mL were started, containing 20 g/L inulin and an enzyme ratio of 269.3 U/g_inulin_, as the results from the hydrolysis experiments showed that further increases in enzyme concentration did not result in significant efficient improvements. The acetate buffer solution and inulin powder were sterilized separately to avoid microbial contamination and inulin degradation. The hydrolysis reaction was conducted at 50 °C, pH 5, and 250 rpm. After 40 min of hydrolysis, 5 mL of a 10 × mineral salt solution was added to the hydrolysates to reach the nutrient levels found in the mineral medium previously described. Then the pH was adjusted to 6.8 using the 4 M NaOH solution, as mentioned in the previous section. Afterward, the media was inoculated using 5 mL from the seed culture, and the fermentation conditions were maintained as described for the seed culture. Samples were taken periodically to measure biomass, carbohydrate, and PHA concentrations. Experiments were run during 72 h, based on *C.* *necator* H16 accumulation reports, which do not report a significant increase in accumulation after 60 h, under batch conditions [23]. For comparison, parallel fermentations from the same seed culture were performed with the mineral medium described added with 15 g/L fructose as the carbon source.

Finally, a control experiment was carried out to corroborate the confluence of the saccharification and fermentation processes to determine the enzyme activity at the fermentation conditions, pH 6.8 and 30 °C. The concentration of inulin was set at 10 g/L for this study to reflect that of the hydrolyzed medium while avoiding possible product inhibition effects. The hydrolysis reaction was followed during a period equivalent to that of the fermentation stage, 72 h. Also, to verify a possible enzyme inactivation during the fermentation period, inulinase was incubated for 72 h under fermentation conditions without substrate; after this, inulin (10 g/L) was added to the medium to verify the saccharification conversion.

### Carbohydrate measurement

Samples obtained during the hydrolysis and fermentation stages were analyzed by the dinitrosalicylic acid (DNS) method to determine the amount of reducing sugars in the media [24]; a calibration curve was constructed using fructose. Additionally, the different carbohydrates, fructose, and glucose were quantified by high-performance liquid chromatography (HPLC). A Unison UK-Amino UKA26 column, 250 × 2 mm, was used with mobile phase acetonitrile:water 85:15 (v:v) at 0.2 mL/min and 50 ºC. An evaporative light scattering detector was coupled to the HPLC, with the temperature of the nebulizer and evaporator set to 50 and 80 °C, respectively, and a nitrogen flow of 1 mL/min.

### Dry matter determination

Samples (1 mL) from the fermentation stage were centrifuged at 9000 rpm for 10 min using pre-weighed micro test tubes. The supernatant was separated for further analysis, and the biomass pellet was dried at 70 °C for 48 h, after which the total biomass was determined gravimetrically.

### PHA content

Dry matter samples were subsequently used for PHA quantification. After drying, samples were ground using a mortar until at least 0.1 g of a fine powder was produced; then, chloroform was added in a 1:10 (w/v) ratio at 60 °C for 1 h to extract the PHA. The hydrophilic fraction of biomass was separated using a 5 × water volume and a separation funnel. The PHA-rich fraction was dried overnight to recover the polymer and weighed to determine PHA content.

### Molecular weight determination

The weight average molecular weight (*M*_w_) of the PHA was determined after 72 h of fermentation by gel permeation chromatography (GPC) using two PLGEL 10 μm MIXED-B columns, 300 mm in length, in series (Varian, Polymer Labs), in an HPLC system with a refractive index detector (Agilent Infinity, 1260). Chloroform was used as the mobile phase at 0.8 mL/min and a column temperature of 50 °C. A calibration curve was constructed using known molecular weight polystyrene standards (from 2.94 × 10^3^ to 2.95 × 10^6^ Da). Samples were dissolved in chloroform at 1–2 mg/mL and filtered through a 0.45 μm filter before injection. The injection volume was set at 40µL for each analysis.

### Nuclear magnetic resonance

Liquid hydrogen NMR analyses were conducted on the extracted PHA to determine the biopolymer structure using a Bruker 500 MHz AVANCE III NMR spectrometer (Bruker GmbH, Germany) with a 5-mm TCI cryoprobe. Deuterated chloroform (99.96%, Sigma-Aldrich) was used as a solvent. 1H NMR spectra were recorded at 303 K using a recycle delay of 12 s, 64 K fid size, and 64 scans.

### Thermal analyses

Differential scanning calorimetry (DSC) and thermogravimetric (TGA) analyses were used to determine the thermal properties of the products obtained. For DSC, samples were heated from − 10 to 200 °C, followed by a cooling stage reaching − 10 °C; finally, samples were then reheated to 200 °C (heating and cooling rates were maintained at 10 °C/min during all stages); a nitrogen flux of 20 mL/min was used for the analyses (Mettler Toledo, model DSC1). The degree of crystallinity, ${\chi }_{\mathrm{c}}$, of a semi-crystalline material melting in the evaluated temperature range using the following equation:

$${\chi }_{\mathrm{c}}= \frac{{\Delta H}_{\mathrm{m}}}{{\Delta H}_{100\%}}100\%,$$

where ${\Delta H}_{\mathrm{m}}$ is the measured heat of fusion (J/g); and ${\Delta H}_{100\%}$ represents the heat of fusion of a 100% crystalline material (146 J/g) [25]. The reusability of the materials was studied through a deterioration analysis based on eight subsequent heating–cooling cycles using similar conditions to those of DSC. TGA experiments were carried out from 30 to 600 °C, with a heating rate of 10 °C/min, under a nitrogen atmosphere (20 mL/min) (Perkin Elmer, model TGA 4000). Temperatures at the maximum degradation rate (*T*_max_) for each sample were determined from the first derivatives of the TGA curves.

## Results and discussion

### Enzymatic hydrolysis of inulin

**Fig. 1:**
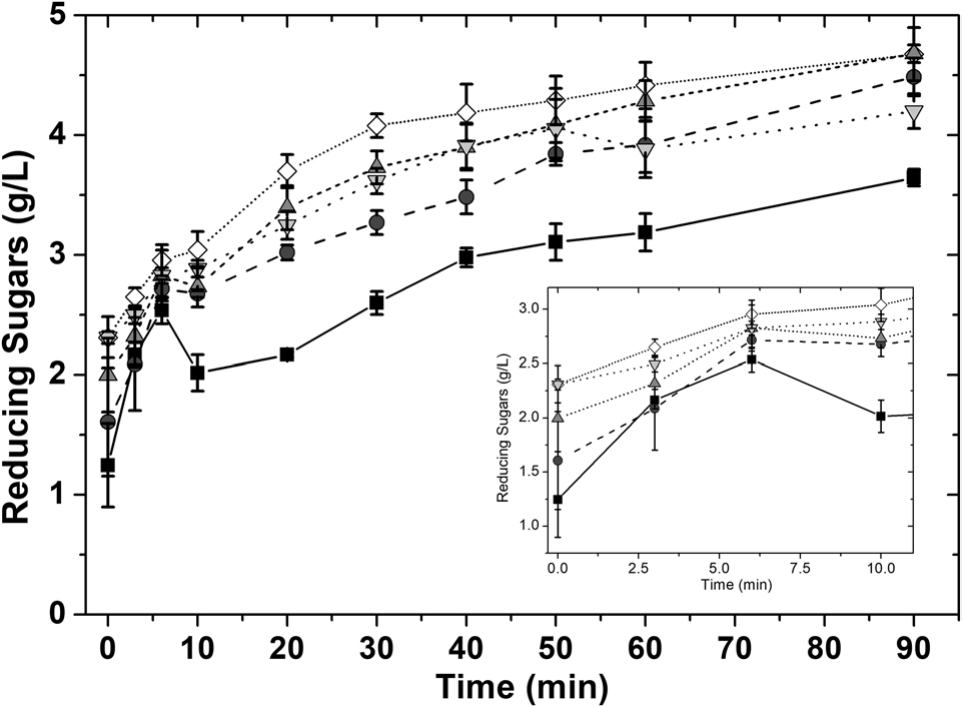
Effect of the enzyme concentration on the hydrolysis of chicory root inulin; using Novozym 960 inulinase at 88 (squares), 180 (circles), 269 (down-pointing triangle), 359 (up-pointing triangle), and 449 U/g_inulin_ (diamonds); at pH 5.0 50 °C and 5 g/L of inulin

Different enzyme concentrations were tested at the reported optimum conditions for inulin hydrolysis by the commercial inulinase Novozym 960 (50 °C, pH 5) [26, 27]. Results show that an inulin conversion yield of approximately 80% can be reached after 40 min (Fig. 1) using an initial inulin concentration of 5 g/L with an enzyme ratio of 180 U/g_inulin_. Comparable results were obtained for the evaluated enzyme concentration interval, from 1 to 5% (v/v), corresponding to an enzyme ratio of 88, 180, 269, 359, and 449U/g_inulin_, respectively.

Markedly, after a reaction time of 90 min, the complete hydrolysis of inulin was not reached for the evaluated conditions, with a maximum inulin conversion measured of 95%, similar to what was found by Corrado et al*.* (2021) who employed *Penicillium* *lanosocoeruleum* inulinases; they reported up to 96% inulin is converted in fructose within 20 h [28]. However, the kinetic profile for fructose released was similar for all enzyme concentrations tested, with most of the substrate hydrolyzed within the first 40 min of reaction, allowing an important reduction in time, needing approximately 8 h to process the same amount of inulin reported by Corrado et al*.*, 60 g/L, with a potential improvement of the process productivity.

**Fig. 2:**
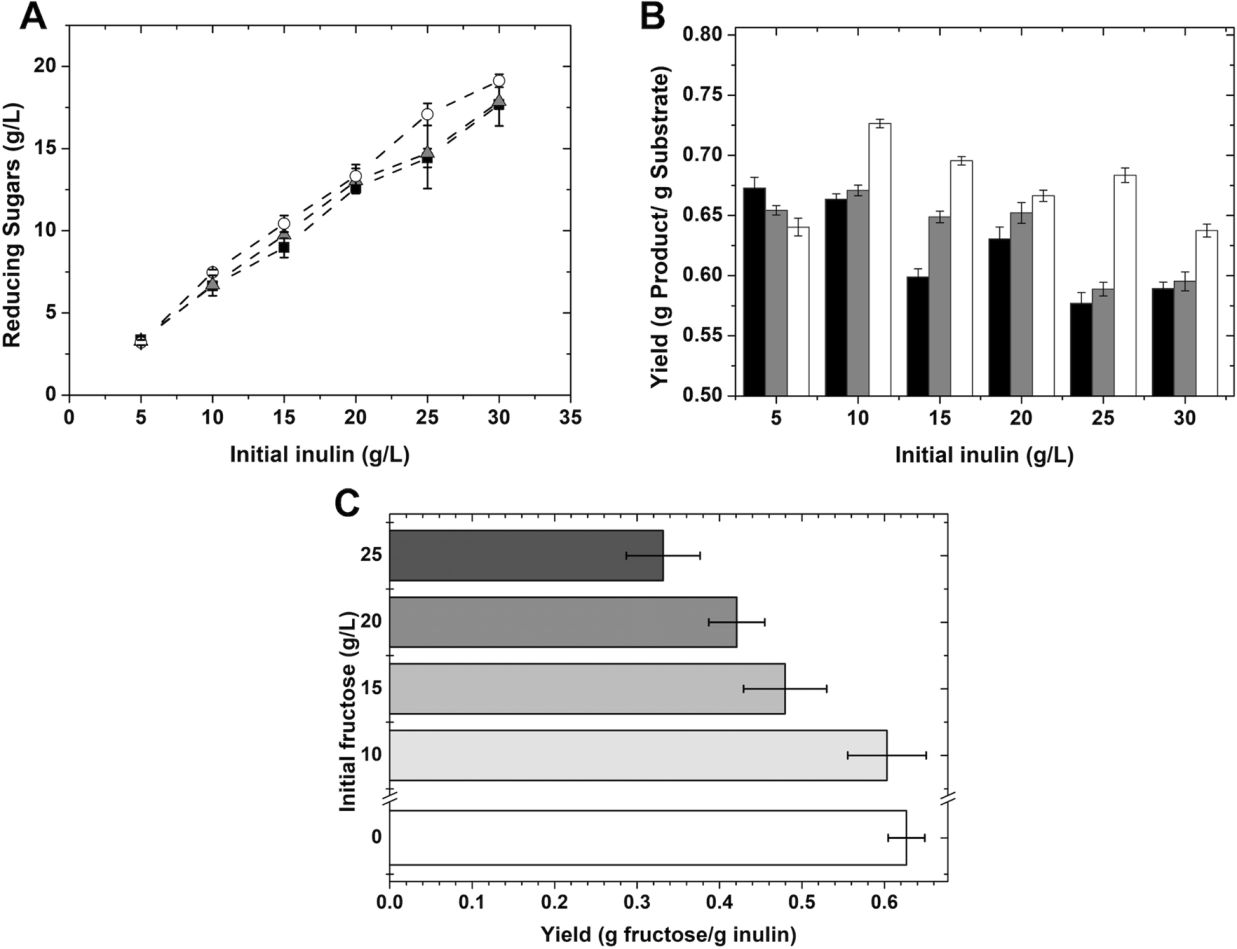
Evaluation of the initial substrate concentration effect on the hydrolysis yield. **a** Reducing sugar concentration after (black squares) 20, (gray triangles) 30, and (open circles) 40 min at different initial inulin concentrations. **b** Hydrolysis product yields obtained at different inulin concentrations (20 min, black; 30 min, gray; 40 min, white). **c** Effect of Initial fructose concentration on the product yield for the enzymatic hydrolysis reaction. All reactions were carried out at pH 5.0 50 °C

To increase the final fructose concentration, several initial inulin concentrations (5, 10, 15, 20, 25, and 30 g/L) were tested at a fixed enzyme ratio of 269.28 U/g_inulin_. Three different reaction times were used: 20, 30, and 40 min (Fig. 2a). A higher concentration of reducing sugars was obtained for each increase in initial inulin concentration. Nevertheless, a drop in yield was measured for initial inulin concentrations above 15 g/L (Fig. 2b). The maximum final fructose concentration of around 18 g/L was reached using an initial inulin concentration of 30 g/L, corresponding to only a 60% (w/w) yield, this low yield can be attributed to a product inhibition effect, agreeing with several reports on inulinases end-product inhibition. Vullo et al*.* reported inulinase inhibition by fructose concentrations as low as 14 mM [29]. Similarly, Mutanda et al*.* determined the reversible competitive inhibition of an exoinulinase from *Aspergillus* *ficuum*, with an increase of almost 50% in the K_m_ value when a 5% initial fructose was added to the reaction medium [30].

The effect of fructose concentration on the hydrolysis yield was measured by adding different fructose concentrations at the beginning of the hydrolysis, with initial inulin and enzyme concentrations of 10 g/L and of 3% (v/v), respectively. The reactions were stopped after 40 min. The results obtained show that initial fructose of 10 g/L has a small negative effect on enzyme activity and reaction yields, around 5% of reduction. Higher concentrations up to 25 g/L led to a 30% or more reduction in yield, compared to the yield obtained when no fructose was added to the system (Fig. 2c), highlighting the importance of fructose removal from the system to avoid product inhibition and reduced yields. The latter can be achieved by introducing a coupled saccharification and fermentation process, where a microorganism consumes the fructose produced, thus avoiding the fructose inhibition effect [28]. Nevertheless, even when the hydrolysis system is less efficient at higher inulin levels, a high concentration of the carbon source is required to trigger PHA synthesis in *C.* *necator*; for instance, a 15 g/L fructose threshold has been proposed [31, 32]. Thus, to ensure that the carbon source concentration necessary for PHA accumulation is attained, the initial inulin concentration was set to 20 g/L for the saccharification–fermentation experiments.

### PHA production by *C. necator* using the hydrolysate media

To determine the ability of *C.* *necator* to grow on hydrolyzed inulin and synthesize PHA in the medium produced, first, a hydrolysis stage using Novozym 960 was carried out under the best conditions, 50 °C and pH 5 as reported elsewhere [4]. Initial inulin and enzyme concentrations were set at 20 g/L and 269.3 U/g_inulin,_ respectively, with a working volume of 50 mL.

To avoid Novozym 960 enzyme denaturation and to continue the enzymatic hydrolysis of inulin after inoculation, the reaction media was not sterilized after the hydrolysis; thus, the system was only maintained under aseptic conditions after the media preparation. This could provide major cost reductions, as it has been suggested that the necessity to maintain sterility conditions elevates PHB production costs for single-cell cultures, reducing the energy requirements related to temperature sterilization [33]. To check for microbial contamination, agar plates with the short-term storage medium were inoculated using the fermentation samples collected and then incubated overnight. The colony morphology was compared to the cell stock, with no contamination found throughout the experiments.

**Fig. 3:**
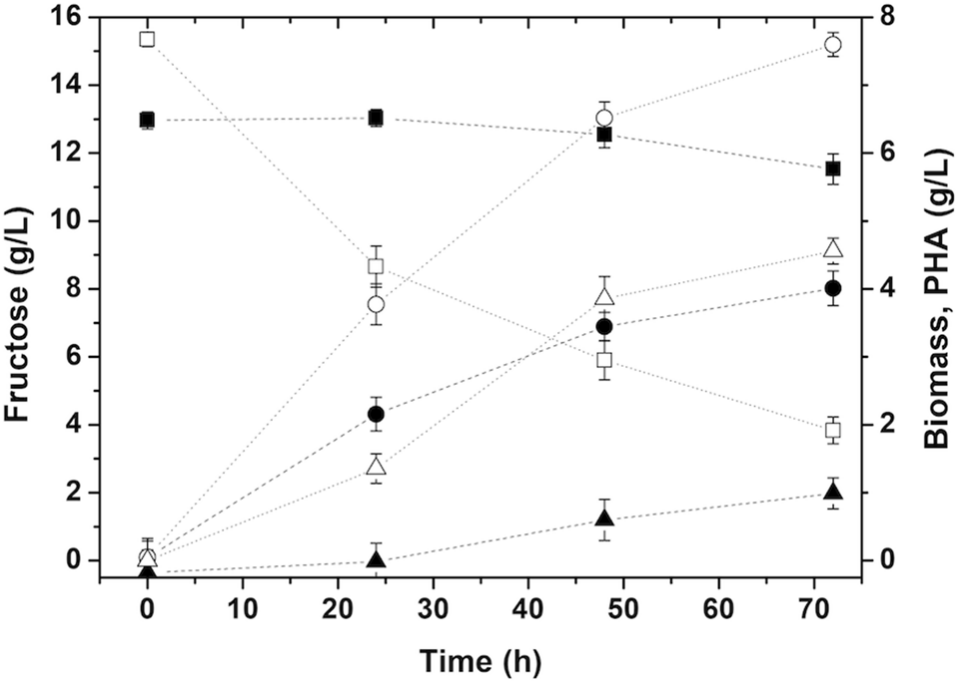
*C.* *necator* cell growth kinetics and polymer accumulation using an enzymatic inulin hydrolysate derived medium (solid symbols), and a fructose-based defined medium (open symbols); at 250 rpm at 30 °C. Squares, fructose concentration (g/L); circles, biomass concentration (g/L); triangles, PHB concentration (g/L)

Fructose concentration results of the hydrolysis stage, in 50 mL of total volume, show that about 70% of the inulin was hydrolyzed after 1 h, and dry-cell matter data demonstrated that *C.* *necator* was able to grow under the conditions provided by the hydrolyzed inulin. A biomass concentration of around 4 g/L was reached, with a PHA accumulation of 30% (w/w) after 72 h (Fig. 3). Markedly, the increase in biomass concentration during the first 24 h of the experiment did not correspond to any decrease in the fructose concentration in the system, suggesting a simultaneous saccharification and fermentation process occurring during the first stage of the process. Corrado et al*.*, studied the feasibility of a similar SSF system using *P.* *lanosocoeruleum* inulinases to convert inulin to PHA by the fructose fermentation using *C.* *necator*, reporting a maximum biomass concentration of 4 g/L with an 82% polymer content after the optimization of the system [28]. The biomass results obtained in this research are comparable to those reported; nevertheless, the polymer accumulation levels reported for the systems were notably different, 30 versus 80% (w/w).

An optimized synthetic medium-based experiment with 15 g/L of fructose as the sole carbon source was used for comparison with the hydrolyzed system [22]. Results led to a higher biomass concentration, 7.6 g/L, with a polymer accumulation of 60% (w/w), under the abovementioned conditions. The lower concentrations achieved from the hydrolysate medium in this work may be due to the introduction of new chemical species, like acetate salts and enzyme impurities, needed for the hydrolysis step. Control experiments using the defined medium, one with added acetate salts and another using the enzyme as the only nitrogen source, demonstrated that *C.* *necator* grows to a similar biomass titer, 4.7 g/L when the enzyme was the only nitrogen source available. A negative effect on microbial growth was observed by adding 0.05 M of acetate salts to the medium, 2.7 g/L. This underscores the importance of nutrient optimization as it has been proven that a high C:N ratio favors polymer synthesis, with the metabolic pathway for PHA is triggered in *C.* *necator* by a combination of high-carbon source concentration and a lower level of a macronutrient, generally nitrogen [34]. Importantly, the strain’s preference for a mineral nitrogen source would avoid the consumption of the enzyme for cell growth, favoring the maintenance of the enzymatic activity.

Nevertheless, it has been proved that the use of purified carbon sources, i.e., fructose, increases PHA production costs, hindering competitivity against petroleum-based plastics and causing the interest in finding new low-cost available raw materials for the production process [10]. Inulin-rich by-products derived from agro-industrial processing represent a cheaper raw material alternative to fructose [1].

**Fig. 4:**
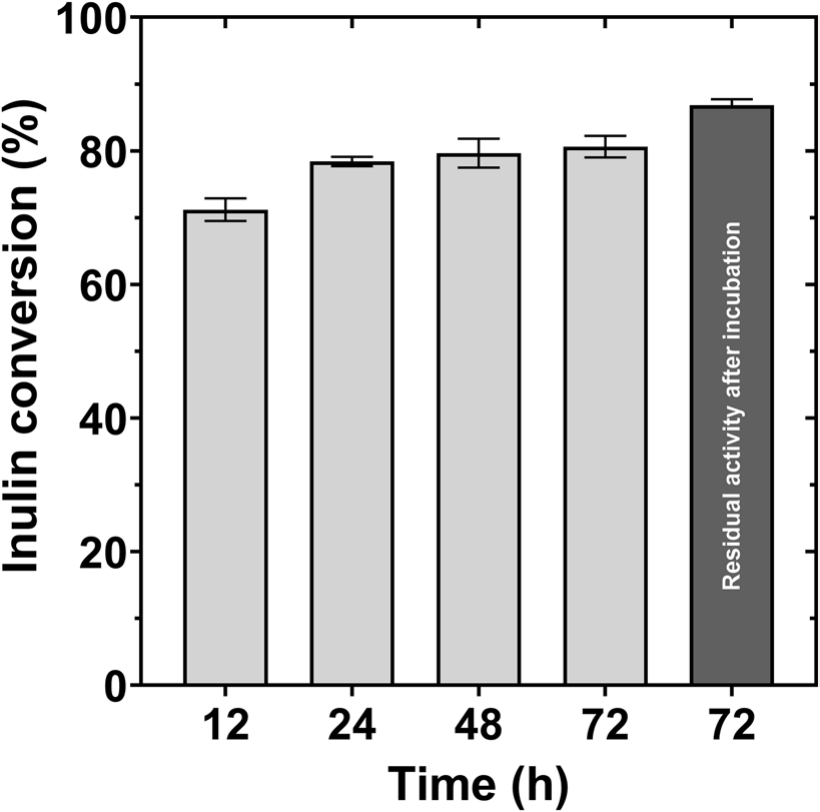
Inulin conversion under fermentation conditions at different times (light gray) and residual activity after incubation (dark gray), at 30 °C, pH 6.8 during 72 h

Hydrolysis experiments performed at fermentation conditions (pH 6.8 and 30 °C) showed that the enzyme hydrolysis reaches 80% (w/w) yield after 24 h, and no increment of fructose concentration was detected afterward, during the 72 h of the experiment (Fig. 4). Even though the direct conversion of inulin into fructose after 24 h has been reported up to a 95% yield under optimal conditions [4], the yield obtained in this experiment can be attributed to the conditions under which it was conducted, far from the hydrolysis optimal conditions. Finally, the incubation of the enzyme under the fermentation conditions for a period equivalent to the fermentation stage revealed that the residual activity remained at 86% of that measured for the fresh enzyme, confirming the convergence of the conditions necessary for the hydrolysis and fermentation, albeit the enzyme is working under non-ideal conditions.

### HNMR and molecular weight characterization

The PHB produced from the fructose-based medium (Fa) and a hydrolyzed inulin medium (Hb) were characterized by HNMR. The spectra obtained from both polymers (Fa and Hb) present the characteristic signals of the polyhydroxybutyrate (PHB) standard, corresponding to literature reports focused on *C.* *necator* growth using fructose as the carbon source [35, 36]. Samples from synthetic medium showed only PHB correspondent signals (Shown in supporting information Fig. A.1A). In contrast, spectra resulting from hydrolysate-based samples revealed unidentified signals, suggesting a copolymer synthesis by the assimilation of other carbon sources that would serve as precursors to different repeating units to butyrate [37]. Aramvash et al*.* examined the effect of feeding different co-substrates to *C.* *necator* H16 when fructose was used as the main carbon source at 20 g/L. They found that the addition of 2% (v/v) acetic acid to the medium led to the insertion of a 12% mol fraction of HV units into the polymer produced. Acetate salts from the hydrolysis buffer (50 mM) are present in the fermentation medium used, which explains the presence of HV signals observed at 0.9 and 1.25 ppm (Fig. A.1B). Nevertheless, the thermal analyses of the materials discussed below showed no significant change in the polymer properties indicating only a small insertion of HV units to the polymer chain, which can be increased by the feeding of HV precursors to the fermentation medium [38].

Further characterization of the obtained materials focused on measuring their molecular weight as an indicator of the material quality and chain length homogeneity, which in turn dictates the potential applications of a plastic material [35]. The weight average molecular weight (*M*_w_) was measured by SEC-GPC using a narrow polystyrene standard calibration curve. The Hb polymers showed a *M*_w_ value of 590 kDa for samples taken at 72 h of fermentation and a Polydispersity Index (PDI) of around 1.7, comparable to what has been reported previously for glycerol-based PHB *M*_w_ 550 kDa with a PDI of 2, but below from the values reported from a glucose-based PHB 890 kDa, PDI 2.4, both produced using *C.* *necator* cultures, similar to the commercial PHB offered by Sigma-Aldrich [39]. On the other hand, the Fa sample, also collected after 72 h of fermentation, reported values of *M*_w_ of 429 kDa, with a PDI of 2.2.

Nevertheless, the narrower polydispersity index of the polymers produced from inulin indicates a more consistent chain length; thus, a higher quality material as the processing conditions and potential applications of a polymer are dependent on the polymer size, with a higher M_w_ leading to a more durable material [40]. Furthermore, the molecular weight of PHB synthesized by *C.* *necator* has shown to be time-dependent, with a 100 kDa reduction from 20 to 40 h fermentation, allowing control of this material property by controlling the biomass-harvesting time if necessary [35, 39].

### Thermal characterization

The thermal characterization of polymers was used to give insight into their processing conditions and qualify their material stability to temperature [41]. DSC and TGA were used to determine the thermal properties of Fa and Hb polymers. Table 1 lists the thermal properties that were measured with DSC. Interestingly, the glass transition temperature (*T*_g_) and degree of crystallinity (*X*_c_) measured for the Fa sample were smaller (1.71 °C, 34.12%) than those obtained for the hydrolysate-based sample, resulting in a more brittle material derived from the hydrolysate, Hb reached values of *T*_g_ and *X*_c_ near 2.27 °C and 59.08%, respectively.

**Table 1:** Thermal properties calculated from the DSC curves obtained from the PHB samples synthesized using a fructose-based synthetic medium (Fa); and a hydrolysate-based medium (Hb)

DSC scan	*T*_*g*_ (°C)^a^	*X*_c_ (%)^a^	Cooling scan			2nd Heating scan		
PHB specimen			*T*_onset_ (°C)	*T*_c_ (°C)	*∆H*_c_ (J/g)^a^	*T*_onset_ (°C)	*T*_m_ (°C)^a^	*∆H*_m_ (J/g)^a^
Fa	1.71	34.12	106.89	94.96	44.63	164.48	169.85	53.27
Hb	2.27	59.08	103.95	89.14	67.61	168.42	176.16	87.91

Glass transition temperature (*T*_g_); degree of crystallinity (*X*_c_); crystallization temperature (*T*_c_), crystallization enthalpy (Δ*H*_c_); melting temperature (*T*_m_); melting enthalpy (Δ*H*_m_)

^a^The measured parameters were marked with superscripts to ensure the clarity of the table as an independent entity

The higher crystallinity (*X*_c_) measured for the hydrolysate-based sample could be attributed to the higher PDI obtained for the synthetic-medium-derived materials, as a higher variation in the polymer chain length renders a more amorphous material [40].

**Fig. 5:**
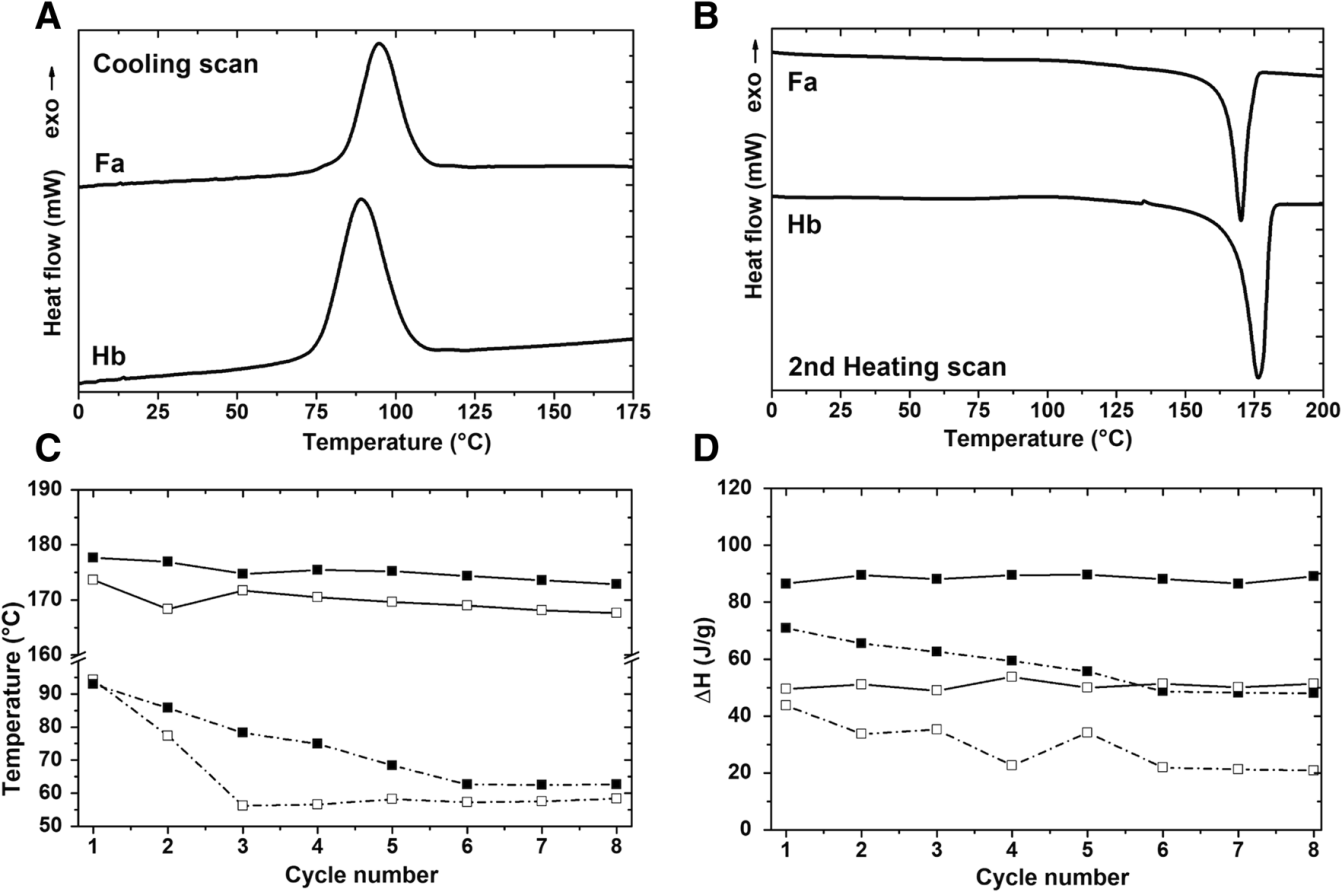
DSC curves obtained from, **A** the cooling process, and (**B**) the second heating scan of PHB samples obtained from 72 h *C.* *necator* cultures grown in, (Fa) a synthetic medium using fructose, and (Hb) an enzymatic hydrolysate medium. Changes on the melting and crystallization temperatures (**C**) and enthalpies (**D**) measured through eight DSC heating and cooling cycles; obtained for PHB samples coming from a fructose-based medium, Fa (open squares); and a hydrolysate-based medium, Hb (closed squares). Solid line: heating scan (melting parameters). Dotted line: cooling scan (crystallization parameters)

The DSC cooling and second heating curves obtained for Fa and Hb were recorded after a heating process necessary to erase the thermal history of the polymers. During the cooling process (Fig. 5a), Fa showed an exothermal crystallization with a value of *T*_c_ of 94.96 °C and a Δ*H*_c_ of 44.63 J/g. Samples Fa and Hb presented a homogeneous crystal size, shown by the presence of a single crystallization peak on each curve. The slight difference found in the *T*_g_ and *T*_m_ temperatures correlates with the *M*_w_ results obtained, with the higher values in polymer size corresponding to higher temperature values obtained by those PHB produced using the hydrolyzed medium. The Hb sample presented a value of *T*_c_ at 89.14 °C and a higher Δ*H*_c_ of 67.61 J/g.

During the second heating stage (Fig. 5b), Fa showed an endothermal fusion peak (*T*_m_) near 169.85 °C and Δ*H*_m_ of 53.27 J/g. Alternatively, Hb exhibits a *T*_m_ value at about 176.16 °C and a higher Δ*H*_m_ of 87.91 J/g. Thus, the thermal stability of the samples can be arranged as Hb > Fa, which corresponds to a higher crystallinity and PDI found for the samples.

To evaluate the potential reusability of polymers Fa and Hb, the thermal behavior of the materials was followed through eight heating–cooling cycles, measuring their melting and crystallization temperatures, as well as their corresponding enthalpies. It is noteworthy that the decreases shown on the *T*_m_ (Fig. 5c) and Δ*H*_m_ (Fig. 5d) values of Hb are less pronounced, over the cycles, with respect to the tendency shown by Fa. Even though both samples showed similar melting and crystallization temperatures at the beginning and at the end of this study, the first and eighth cycles, respectively, the more pronounced drop on the *T*_c_ of the polymer obtained for Fa sample emphasize the higher stability of the polymers derived from the hydrolysate-based medium. If the thermal rupture of the PHB chains is avoided by the addition of another bioplastic, e.g., PLA, or a biodegradable plasticizer, such as maleic anhydride, the potential recycling of these materials represents a significant step towards the circular economy of bioplastics [42].

Finally, the thermal stability of samples Fa and Hb was determined by TGA analysis. The thermograms obtained from the previous materials showed similar behaviors for both samples (Fig. A2). Hb sample experienced a weight loss of 97.11 wt.%. While Fa only loses 71.52 wt.%, an important percentage of this weight loss happens before reaching the decomposition temperature, generally associated with a higher impurities content. Both specimens showed degradation in one step at a temperature between 280 and 327 °C. The onset degradation temperatures of the samples follow the order Fa (284.38 °C) < Hb (286.28 °C).

Nevertheless, both samples reached their maximum degradation rate at the same temperature, 307.20 °C for Hb and 307.16 °C for Fa; these results coincide with those reported by Iglesias Montes et al*.* for a biobased neat PHB sample with a crystallinity of 62% [43]. Aydermir and Gardner, when studying the addition of cellulose nanofibers to biobased PLA/PHB blends, they noticed that, although the effect of cellulose was negligible, PHB is immiscible with PLA, their blend enhances their thermal and rheological behavior, another useful strategy to broaden their field of application without compromising biodegradability [42, 43]. The weight loss shown by Fa suggests the presence of impurities in the sample. Hong et al*.* reported that impurities, such as protein residues, metal compounds like Ca^2+^ and Mg^2+^, and short-chain polymers, greatly influence the thermal stability of the polymers [44], thus stressing the importance of the purification process to attain the desired properties [11, 45].

The results presented here attest to the feasibility of using inulin hydrolysates as a fructose source for PHA production. Further research is needed to optimize the SSF process, focusing on heightening biomass and polymer yields. On the other hand, chicory root inulin is a linear polysaccharide that is readily hydrolyzed by inulinases such as Novozym 960. Other agro-industrial residues, such as agave inulin, a high-branched fructose polymer, resulting in a more complex substrate for commercial enzymes, highlighting the necessity for the search and characterization of novel enzyme activities [21].

## Conclusions

This work demonstrated the feasibility of PHB production from chicory root inulin hydrolysates, enabling a new valorization strategy for inulin-rich by-products towards PHB production. A maximum fructose titration of 18 g/L was attained during the hydrolysis, above the inhibition concentration found for the enzyme, 15 g/L. The inoculation of this medium confirmed the occurrence of simultaneous saccharification–fermentation conditions during the first 24 h of the proposed system, preventing the potential enzymatic inhibition caused by fructose accumulation, resulting in the biomass growth and polymer accumulation of 4 g/L biomass with 30% polymer accumulation. Furthermore, such results serve as an important proof of concept for exploring the simultaneous saccharification–fermentation processing of inulin to produce PHB as a reduction-cost strategy to increase its viability as a plastic replacement. Finally, the biopolymers obtained from the hydrolysate were more homogeneous, in their M_w_, and thermally stable than those produced in a mineral medium, suggesting the potential recycling of the material, a threshold needed to attain the circular economy of bioplastics.

### Supplementary Information

Below is the link to the electronic supplementary material.

Supplementary file1 ^1^HNMR spectra and thermograms were obtained for PHA samples extracted from the two fermentation media tested. (DOCX 408 KB)

## Data Availability

The authors declare that the data supporting the findings of this study are available within the paper and its Supplementary Information files. Should any raw data files be needed in another format, they are available from the corresponding author upon reasonable request.

## References

[1] Rawat HK, Soni H, Treichel H, Kango N (2017). Biotechnological potential of microbial inulinases: recent perspective. Crit Rev Food Sci Nutr.

[2] Apolinário AC, De Lima Damasceno BPG, de Macêdo Beltrão NE, Pessoa A, Converti A, da Silva JA (2014). Inulin-type fructans: a review on different aspects of biochemical and pharmaceutical technology. Carbohydr Polym.

[3] Ávila-Fernández Á, Rendón-Poujol X, Olvera C, González F, Capella S, Peña-Alvarez A, López-Munguía A (2009). Enzymatic hydrolysis of fructans in the tequila production process. J Agric Food Chem.

[4] Singh R, Singh S (2016). Inulinases. Current developments in biotechnology and bioengineering: production, isolation and purification of industrial products.

[5] García-Aguirre M, Sáenz-Álvaro VA, Rodríguez-Soto MA, Vicente-Magueyal FJ, Botello-Álvarez E, Jimenez-Islas H, Cárdenas-Manríquez M, Rico-Martínez R, Navarrete-Bolaños JL (2009). Strategy for biotechnological process design applied to the enzymatic hydrolysis of agave fructo-oligosaccharides to obtain fructose-rich syrups. J Agric Food Chem.

[6] Duvigneau S, Dürr R, Behrens J, Kienle A (2021). Advanced kinetic modeling of bio-co-polymer poly(3-hydroxybutyrate-co-3-hydroxyvalerate) production using fructose and propionate as carbon sources. Processes.

[7] Amache R, Sukan A, Safari M, Roy I, Keshavarz T (2013). Advances in PHAs production. Chem Eng Trans.

[8] Koller M, Braunegg G (2018). Advanced approaches to produce polyhydroxyalkanoate (PHA) biopolyesters in a sustainable and economic fashion. Eurobiotech J.

[9] Nasir Iqbal HM (2012). Economical bioconversion of lignocellulosic materials to value-added products. J Biotechnol Biomater..

[10] Choi J, Lee SY (1999). Factors affecting the economics of polyhydroxyalkanoate production by bacterial fermentation. Appl Microbiol Biotechnol.

[11] Wongsirichot P, Gonzalez-Miquel M, Winterburn J (2019). Holistic valorization of rapeseed meal utilizing green solvents extraction and biopolymer production with *Pseudomonas*
*putida*. J Clean Prod.

[12] Yousuf R, Winterburn J (2016). Date seed characterisation, substrate extraction and process modelling for the production of polyhydroxybutyrate by *Cupriavidus*
*necator*. Bioresour Technol.

[13] Urbina L, Wongsirichot P, Corcuera MÁ, Gabilondo N, Eceiza A, Winterburn J, Retegi A (2018). Application of cider by-products for medium chain length polyhydroxyalkanoate production by *Pseudomonas*
*putida* KT2440. Eur Polym J.

[14] Lee SM, Cho DH, Jung HJ, Kim B, Kim SH, Bhatia SK, Gurav R, Jeon JM, Yoon JJ, Kim W, Choi KY, Yang YH (2022). Finding of novel polyhydroxybutyrate producer Loktanella sp. SM43 capable of balanced utilization of glucose and xylose from lignocellulosic biomass. Int J Biol Macromol.

[15] Peña-Jurado E, Pérez-Vega S, Zavala-Díaz de la Serna FJ, Pérez-Reyes I, Gutiérrez-Méndez N, Vazquez-Castillo J, Salmerón I (2019). Production of poly (3-hydroxybutyrate) from a dairy industry wastewater using Bacillus subtilis EPAH18: bioprocess development and simulation. Biochem Eng J.

[16] Leong YK, Show PL, Lan JCW, Loh HS, Lam HL, Ling TC (2017). Economic and environmental analysis of PHAs production process. Clean Technol Environ Policy.

[17] Penloglou G, Vasileiadou A, Chatzidoukas C, Kiparissides C (2017). Model-based intensification of a fed-batch microbial process for the maximization of polyhydroxybutyrate (PHB) production rate. Bioprocess Biosyst Eng.

[18] Ferre-Guell A, Winterburn J (2017). Production of the copolymer poly(3-hydroxybutyrate-*co*-3-hydroxyvalerate) with varied composition using different nitrogen sources with Haloferax mediterranei. Extremophiles.

[19] Verlinden R, Hill D, Kenward M, Williams C, Radecka I (2007). Bacterial synthesis of biodegradable polyhydroxyalkanoates. J Appl Microbiol.

[20] Koller M (2018). Martin, biodegradable and biocompatible polyhydroxy-alkanoates (PHA): auspicious microbial macromolecules for pharmaceutical and therapeutic applications. Molecules.

[21] Trapala J, Bustos-Jaimes I, Manzanares P, Bárzana E, Montiel C (2020). Purification and characterization of an inulinase produced by a Kluyveromyces marxianus strain isolated from blue agave bagasse. Protein Expr Purif.

[22] Aramvash A, AkbariShahabi Z, DashtiAghjeh S, Ghafari MD (2015). Statistical physical and nutrient optimization of bioplastic polyhydroxybutyrate production by *Cupriavidus*
*necator*. Int J Environ Sci Technol.

[23] Khan M, Prasad D, Abdullah H, Batcha A (2013). Kinetic analysis on cell growth and biosynthesis of poly (3-hydroxybutyrate) (PHB) in *Cupriavidus*
*necator* H16. Int J Biosci Biochem Bioinforma..

[24] Marsden WL, Gray PP, Nippard GJ, Quinlan MR (1982). Evaluation of the DNS method for analysing lignocellulosic hydrolysates. J Chem Tech Biolechnol.

[25] Barham PJ, Keller A, Otun EL, Holmes PA (1984). Crystallization and morphology of a bacterial thermoplastic: poly-3-hydroxybutyrate. J Mater Sci.

[26] Kango N, Jains SC (2011). Production and properties of microbial inulinases: recent advances. Food Biotechnol.

[27] Singh RS, Singh T, Pandey A (2019). Microbial enzymes-an overview. Biomass, biofuels, biochemicals: advances in enzyme technology.

[28] Corrado I, Cascelli N, Ntasi G, Birolo L, Sannia G, Pezzella C (2021). Optimization of inulin hydrolysis by penicillium lanosocoeruleum inulinases and efficient conversion into polyhydroxyalkanoates. Front Bioeng Biotechnol.

[29] Vullo DL, Coto CE, Sineriz F (1991). Characteristics of an inulinase produced by Bacillus subtilis 430A, a strain isolated from the rhizosphere of Vernonia herbacea (Vell Rusby). Appl Environ Microbiol.

[30] Mutanda T, Wilhelmi B, Whiteley CG (2009). Controlled production of fructose by an exoinulinase from aspergillus Ficuum. Appl Biochem Biotechnol.

[31] Cavalheiro J, de Almeida M, da Fonseca M, de Carvalho C (2012). Adaptation of *Cupriavidus*
*necator* to conditions favoring polyhydroxyalkanoate production. J Biotechnol.

[32] Nygaard D, Yashchuk O, Noseda DG, Araoz B, Hermida ÉB (2021). Improved fermentation strategies in a bioreactor for enhancing poly(3-hydroxybutyrate) (PHB) production by wild type *Cupriavidus*
*necator* from fructose. Heliyon.

[33] Serafim LS, Lemos PC, Albuquerque E, Reis M (2008). Strategies for PHA production by mixed cultures and renewable waste materials. Appl Microbiol Biotechnol.

[34] Shahid S, Mosrati R, Ledauphin J, Amiel C, Fontaine P, Gaillard JL, Corroler D (2013). Impact of carbon source and variable nitrogen conditions on bacterial biosynthesis of polyhydroxyalkanoates: evidence of an atypical metabolism in Bacillus megaterium DSM 509. J Biosci Bioeng.

[35] Pradhan S, Dikshit K, Moholkar VS, Pradhan S, Dikshit PK, Moholkar VS (2020). Production, characterization, and applications of biodegradable polymer: polyhydroxyalkanoates.

[36] Dalsasso RR, Pavan FA, Bordignon SE, de Aragão GMF, Poletto P (2019). Polyhydroxybutyrate (PHB) production by *Cupriavidus*
*necator* from sugarcane vinasse and molasses as mixed substrate. Process Biochem.

[37] Anjum A, Zuber M, Zia KM, Noreen A, Anjum MN, Tabasum S (2016). Microbial production of polyhydroxyalkanoates (PHAs) and its copolymers: a review of recent advancements. Int J Biol Macromol.

[38] Aramvash A, Hajizadeh-Turchi S, Moazzeni-zavareh F, Gholami-Banadkuki N, Malek-sabet N, Akbari-Shahabi Z (2016). Effective enhancement of hydroxyvalerate content of PHBV in *Cupriavidus*
*necator* and its characterization. Int J Biol Macromol.

[39] Tanadchangsaeng N, Yu J (2012). Microbial synthesis of polyhydroxybutyrate from glycerol: gluconeogenesis, molecular weight and material properties of biopolyester. Biotechnol Bioeng.

[40] Gilbert M (2017). Relation of structure to chemical properties. Brydson’s plastics materials.

[41] Carrasco F, Dionisi D, Martinelli A, Majone M (2006). Thermal stability of polyhydroxyalkanoates. J Appl Polym Sci.

[42] Aydemir D, Gardner DJ (2020). Biopolymer blends of polyhydroxybutyrate and polylactic acid reinforced with cellulose nanofibrils. Carbohydr Polym.

[43] Iglesias Montes ML, D’amico DA, Manfredi LB, Cyras VP (2019). Effect of natural glyceryl tributyrate as plasticizer and compatibilizer on the performance of bio-based polylactic acid/poly(3-hydroxybutyrate) blends. J Polym Environ.

[44] Hong SG, Lin YC, Lin CH (2008). Crystallization and degradation behaviors of treated polyhydroxybutyrates. React Funct Polym.

[45] Madkour H, Heinrich D, Alghamdi A, Shabbaj I, Steinbüchel A (2013). PHA recovery from biomass. Biomacromol.

