# Supplementary material for: Production of polyhydroxybutyrate by coupled saccharification–fermentation of inulin
Source: Bioprocess Biosyst Eng. 2023 Nov 25;47(1):119–29. doi: 10.1007/s00449-023-02953-7 (PMC10776465; doi:10.1007/s00449-023-02953-7)
Supplement: Supplementary file 1 — Supplementary file1 1HNMR spectra and thermograms were obtained for PHA samples extracted from the two fermentation media tested. (DOCX 408 KB) [file 449_2023_2953_MOESM1_ESM.docx]

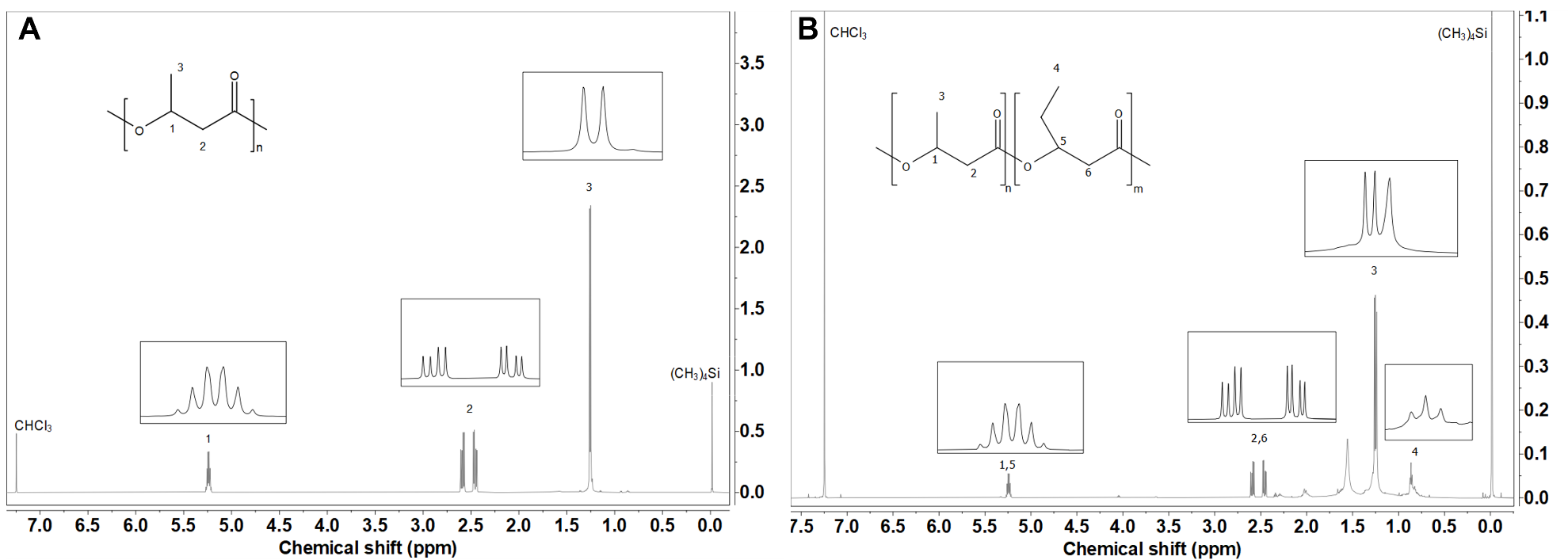


**Fig. A.1.** HNMR spectra of PHA obtained from 72 h *C. necator* cultures grown using a fructose-based synthetic medium using fructose (A); and an enzymatic hydrolysate medium (B).


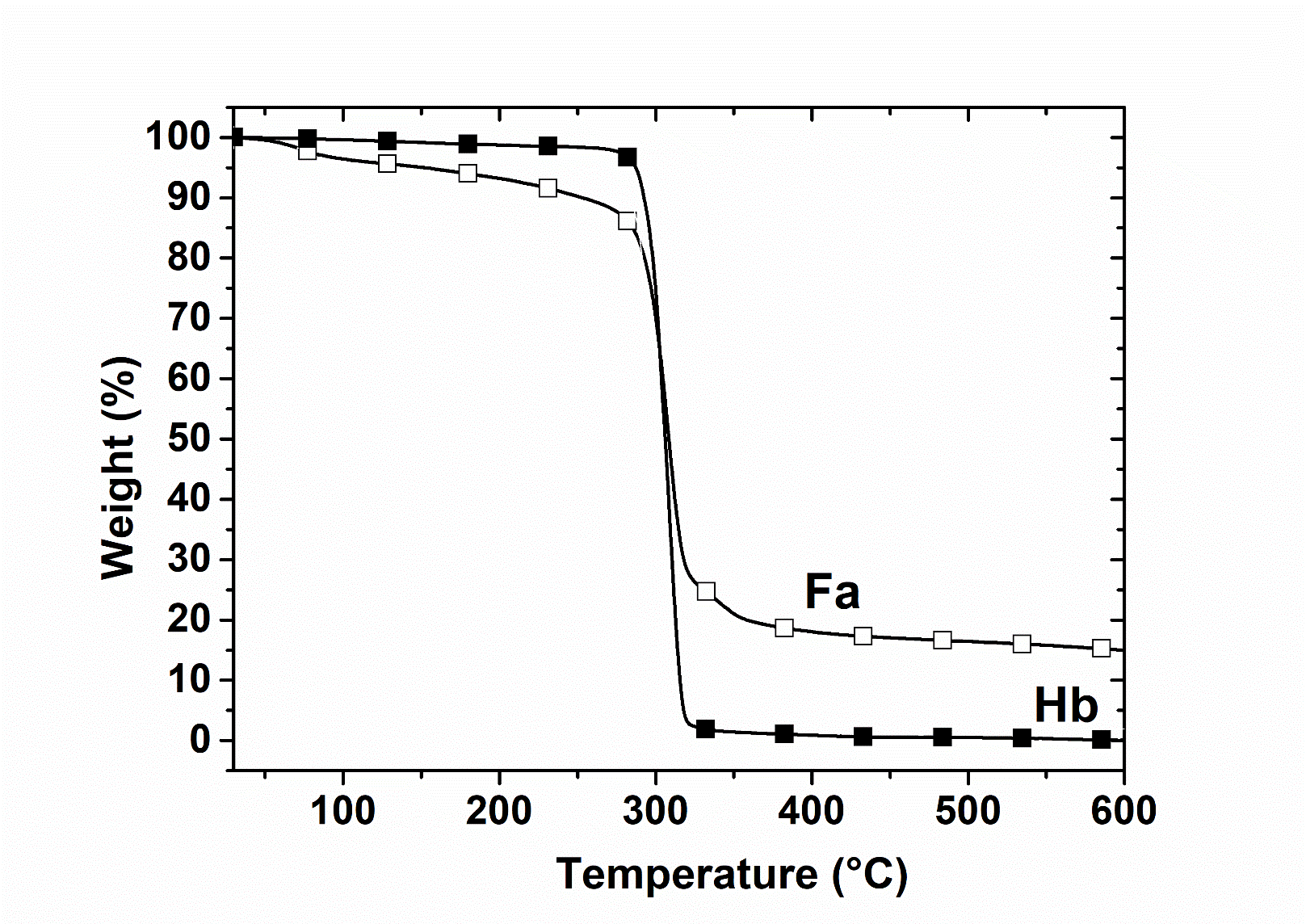


**Fig. A.2.** Thermograms of the PHB samples obtained from a fructose-based synthetic medium, Fa (open squares), and a hydrolysate-based media, Hb (closed squares).
